# *Panax quinquefolius* polysaccharides ameliorate ulcerative colitis in mice induced by dextran sulfate sodium

**DOI:** 10.3389/fimmu.2023.1161625

**Published:** 2023-06-21

**Authors:** Duo-duo Ren, Ke-Cheng Chen, Shan-shan Li, Yan-ting Zhang, Zhi-man Li, Shuang Liu, Yin-shi Sun

**Affiliations:** ^1^ Institute of Special Wild Economic Animals and Plants, Chinese Academy of Agricultural Sciences, Changchun, China; ^2^ Institute of College of Veterinary Medicine, Northwest Agriculture and Forestry University, Yangling, China; ^3^ Goldenwell Biotech, Inc., Nevada, United States; ^4^ Institute of Biological and Pharmaceutical Engineering, Jilin Agricultural Science and Technology University, Jilin, China; ^5^ Looking Up 9 Starry Sky Medical Research Center, Siping, China

**Keywords:** *Panax quinquefolius* polysaccharides, ulcerative colitis, 16S rRNA gene sequencing, gut microbiota, short-chain fatty acids

## Abstract

This study aimed to investigate the ameliorative effect of the polysaccharides of *Panax quinquefolius* (WQP) on ulcerative colitis (UC) induced by dextran sulfate sodium (DSS) in mice and to explore its mechanism. Male C57BL/6J mice were randomly divided into the control group (C), model group (DSS), positive control mesalazine (100 mg/kg, Y) group, and low (50 mg/kg, L), medium (100 mg/kg, M) and high dose (200 mg/kg, H) of WQP groups. The UC model was induced by free drinking water with 2.5% DSS for 7 days. During the experiment, the general condition of the mice was observed, and the disease activity index (DAI) was scored. The conventional HE staining was used to observe pathological changes in mice’s colon, and the ELISA method was used to detect the levels of interleukin-6 (IL-6), IL-4, IL-8, IL-10, IL-1β and tumor necrosis factor-α (TNF-α) in mice’s colon. The changes in gut microbiota in mice were detected by high-throughput sequencing; the concentration of short-chain fatty acids (SCFAs) was determined by gas chromatography; the expression of related proteins was detected by Western blot. Compared with the DSS group, the WQP group showed a significantly lower DAI score of mice and an alleviated colon tissue injury. In the middle- and high-dose polysaccharides groups, the levels of pro-inflammatory cytokines IL-6, IL-8, IL-1β and TNF-α in the colonic tissue were significantly decreased (*P*<0.05), while the levels of IL-4 and IL-10 were significantly increased (*P*<0.05). The 16S rRNA gene sequencing results showed that different doses of WQP could regulate the composition and diversity of gut microbiota and improve its structure. Specifically, at the phylum level, group H showed an increased relative abundance of *Bacteroidetes* and a decreased relative abundance of *Firmicutes* compared with the DSS group, which was closer to the case in group C. At the family level, the relative abundance of *Rikenellaceae* in L, M and H groups increased significantly, close to that in group C. At the genus level, the relative abundance of *Bacteroides*, *Shigella* and *Oscillospira* in the H group increased significantly, while that of *Lactobacillus* and *Prevotella* decreased significantly. The high-dose WQP group could significantly increase the contents of acetic acid, propionic acid, butyric acid, and total SCFAs. Different doses of WQP also increased the expression levels of tight junction proteins ZO-1, Occludin and Claudin-1. To sum up, WQP can regulate the gut microbiota structure of UC mice, accelerate the recovery of gut microbiota, and increase the content of Faecal SCFAs and the expression level of tight junction proteins in UC mice. This study can provide new ideas for the treatment and prevention of UC and theoretical references for the application of WQP.

## 1 Introduction

Inflammatory bowel disease (IBD) is a non-specific, chronic intestinal inflammatory disease with unknown etiology and mediated by abnormal immunity. This inflammation varies in severity, occurs repeatedly, and can even accompany the patient for life, and it imposes huge financial and psychological burdens on the patient and family (1). IBD mainly includes ulcerative colitis (UC) and Crohn’s disease (CD) (2). CD is a discontinuous lesion throughout the digestive tract characterized by diarrhea, while UC is a continuous lesion gathered in the colon, with bloody stool as a prominent feature. Clinically, UC is often characterized by diarrhea, mucous stool, and purulent and bloody stool. The continuous immune reaction in the intestinal tract damages the intestinal mucosa and submucosa and causes symptoms such as intestinal inflammation, ulcer, erosion, and bleeding. Additionally, UC is usually accompanied by intestinal microbiota and metabolic disorders (3, 4). Since the exact cause of UC is unknown, various current treatments are mainly aimed at relieving symptoms, improving systemic conditions, and reducing colonic inflammation and recurrence. Therefore, the search for stable and safe therapeutic drugs for UC has been the focus of researchers.

It is reported that many factors are related to the occurrence and development of UC (5). Studies have shown that the structure of gut microbiota in patients with UC changes significantly, and gut microbiota can regulate the intestinal micro-ecological environment by acting on metabolites and affecting signaling pathways and immune cytokines (6, 7). The gut microbiota plays a key role in human health, co-exists with the host and participates in important metabolism. In general, when the body is in a healthy state, the levels of pro-inflammatory and anti-inflammatory factors are balanced. Once the balance of the gastrointestinal system is destroyed, physical damage to the intestinal mucosa will occur. A complete intestinal mucosal barrier is critical to resist microbial invasion. Thus, the damage to the system may adversely affect the isolation of intestinal contents and nutrient absorption and even be life-threatening (8, 9).

The treatment of IBD has achieved good results with human monoclonal antibodies and recombinant cytokines, but its clinical application is limited due to high costs and serious side effects (10). In addition, currently approved therapeutic drugs for UC, such as antibiotics, steroids, 5-aminosalicylates, glucocorticoids and immunosuppressants, have certain side effects (11). Natural active extracts are widely available with fewer side effects, making them a safer and more effective substitute. As natural macromolecular compounds, polysaccharides can improve intestinal injury through their structural characteristics and biological activity. There have been many reports suggesting that polysaccharides from natural plants are beneficial to intestinal health; for example, ginseng polysaccharides can improve TLR4/MyD88/NF-κB-induced colitis by inhibiting the activation of dextran sulfate sodium (DSS) (12). Therefore, plant polysaccharides may be used as potential therapeutic or adjuvant active components of UC.

American ginseng (*Panax quinquefolius* L.), the dried root of American ginseng, is a perennial herb of the *Araliaceae* ginseng family, which has the effect of tonifying qi and nourishing yin and clearing heat and promoting fluid (13). Modern pharmacological studies have shown that the polysaccharides of *Panax quinquefolius* (WQP) have various biological activities, such as anti-tumor, anti-inflammation, anti-oxidation, and immune regulation. The previous studies of our group have confirmed that WQP can promote the repair of intestinal tissue structure and improve the gut microbiota structure in mice with antibiotic-associated diarrhea (14). With a certain immunomodulatory effect, WQP can regulate the diversity of gut microbiota (15). It is speculated that WQP has a positive therapeutic effect on UC. Therefore, in this study, we investigated the efficiency of WQP in ameliorating UC and further explored the related mechanism in aspects of the immune response, gut microbiota and short-chain fatty acids (SCFAs) by adopting a DSS-induced UC model in C57BL/6J mice. The present study revealed the potential of WQP in the prevention and treatment of UC and provided a theoretical basis for the research and development of functional WQP products.

## 2 Materials and methods

### 2.1 Materials

Dextran sulfate sodium (DSS, CAS NO.160110) was obtained from MP Biomedicals, LLC. Mesalazine enteric-coated tablets were purchased from Tianhong Pharmaceutical Co., Ltd. (Heilongjiang, China). Mouse interleukin 1β (IL-1β; ml301814), mouse interleukin 6 (IL-6; ml002293), mouse interleukin 8 (IL-8; ml001856), mouse tumor necrosis factor alpha (TNF-α; ml002095), mouse interleukin 4 (IL-4; ml002149), mouse interleukin 10 (IL-10; ml002285) kits were obtained from Shanghai Enzyme-Linked Biotechnology Co., Ltd. (MLBIO). Occludin (E-5; sc-133256), Claudin-1 (A-9; sc-166338), Z0-1 (R40.76; sc-33725), GAPDH (G-9; sc-365062) were obtained from Santa Cruz Biotechnology, Inc. The TIANamp Stool DNA Kit (DP328) was obtained from Tiangen Biotech Co., Ltd. (Beijing, China). The other reagents used in the present study were brought from Sinopharm Chemical Reagent Beijing Co., Ltd.

The water-soluble polysaccharides of *Panax quinquefolius* (WQP) was extracted from the research group. Specifically, the dried roots of *Panax quinquefolius* (500 g) cut into small pieces of 1 centimeter, and suspended in 8 L of distilled water for 2 h and heated at 100°C for 4 h, then 120 mesh gauze filter. The resulting filter residue was re-extracted twice, and each time 2 h, combined with the resulting extract, concentrated to 1.5 L using a single frying machine. The concentrated solution was centrifuged (4500 rpm, 10 min), the supernatants were taken. Four times the volume of anhydrous ethanol was added to the supernatants to precipitate it, and then it was left standing for more than 6 h, centrifuged (4500 rpm, 10 min), and the precipitates were taken. The precipitates were dissolved in 800 mL of distilled water, and then centrifuged (4500 rpm, 10 min), the supernatants were taken again. Four times the volume of anhydrous ethanol was added to the supernatant to precipitate it, and it was left standing for more than 12 h, then centrifuged (4500 rpm, 10 min), the precipitates were taken. The precipitations were dissolved in 800 mL of distilled water, Sevag reagent (Chloroform: n-butylalcohol = 4:1, v:v) was used three times to remove the protein layer. The polysaccharides solution layer was collected, and anhydrous ethanol was added to the final concentration of 80% ethanol, then centrifuged (4500 rpm, 10 min), the precipitations were taken and vacuum freeze-dried to yield water-soluble polysaccharides from *Panax quinquefolius* (WQP) were obtained. According to the results of previous experiments, the yield of WQP is 6.71% (w/w). All carbohydrates, uronic acids and protein contents are 85.2%, 31.9% and 2.1%, respectively. It is mainly composed of glucose (33.2%), galactose (8.9%), arabinose (12.2%), galacturonic acid (43.9%), and a small amount of rhamnose (1.8%) (14).

### 2.2 Animal ethical statement

A total of 48 healthy SPF male C57BL/6J mice aged 6–8 weeks were purchased from Huafukang Biotechnology Co., Ltd. (Beijing, China). Animal production license No. SCXK (Beijing, China) 2019-0008 and qualified number No.1103222101975967 were obtained. Male mice were housed at 22–24 °C with 50–60% humidity and a cycle of 12 h light/12 h dark. The animals were given unlimited access to diet and water in the laboratory. These mice were cared for in accordance with the Guidelines for the Care Use of Laboratory Animals, encouraged by the Chinese Legislation on Laboratory Animals, the Chinese Academy of Agricultural Sciences and the Institute of Special Animal and Plant Science. The study was approved by the Experimental Animal Ethics Committee of the Institute of Specialties, Chinese Academy of Agricultural Sciences (Changchun, China) [NO. ISAPSAEC-2021-61M]. All efforts were made to maximize the protection of mice, alleviate their pain, and minimize the total number of mice in the experiment.

### 2.3 *In vivo* experiment design

After 7 days of adaptation to the experimental environment, 48 mice were randomly divided into 6 groups (*n*=8/group): normal control group (C), UC group (DSS), positive control mesalazine group (Y), and low (L), medium (M) and high (H) dose of WQP groups. The mice in DSS, Y, L, M and H groups were given 2.5% DSS daily for 7 days, while group C was given an equal amount of normal saline. According to The Chinese Pharmacopoeia (2020 Edition), the intragastric dose was converted from 6 g/60 kg daily dose of human body, and the low-dose of WQP was selected 50 mg/kg/day in mice, while the medium-dose and high-dose was 100 mg/kg/day and 200 mg/kg/day respectively, based on the preliminary experiment.

From the 8th day, mice in group Y were given mesalazine (100 mg/kg/day), group L was given WQP (50 mg/kg/day), group M was given WQP (100 mg/kg/day), and group H was given WQP (200 mg/kg/day) once a day for 7 days. Group C and group DSS were given an equal amount of normal saline. Fecal samples were taken under aseptic conditions 12 hours after the last administration, frozen in liquid nitrogen and stored at -80°C. At the end of the experiment, eye frame blood was collected for each group of mice, and the blood of the mice was centrifuged at 3000 rpm for 15 min at 4°C, and stored at -80°C. Then the mice were dissected, and the length of the colon from the anus to the root of the cecum was measured for the following experiments. Then, the colon was rinsed with precooled saline, and the colon segment was taken at the same position. The posterior segment of the colon was fixed in a 10% neutral formalin solution, and the tissue of the anterior segment of the colon was stored in a refrigerator at -80°C.

### 2.4 General observation

During the experiment, the body weight, stool characteristics and occult blood status of mice were observed and recorded daily. The disease activity index (DAI) was used for scoring, and the scoring criteria are shown in **Table 1** (16).

**Table 1:** DAI score criterion.

Scores	Weight loss/(%)	Stool consistency	Stool occult blood
0	–	Normal stool	Blood negative (-)
1	1–5	Soft stool	Blood positive (±)
2	5–10	Loose stool	Stool with slight blood (+)
3	10–15	Unformed stool	Stool with blood (++)
4	> 15	Watery diarrhea	Stool with severe blood (>++)

The final score is the average of the sum of the three items.

### 2.5 Histological analysis

The colon tissue was fixed in 10% neutral formalin solution, dehydrated, embedded in paraffin, trimmed and cut into 4–5 μm thick sections, which were stained with conventional HE. The injury of colon tissue was observed under a microscope and photographed.

### 2.6 Determination of cytokines

The colonic tissues of mice in each group were cut and weighed, and the corresponding PBS was added according to the weight. The tissue samples were homogenized fully with a homogenizer and centrifuged (3000 rpm/min, 20 min) at 4°C. The supernatant was collected carefully and stored in an EP tube. Measurement of cytokine was performed using ELISA kits according to the manufacturer’s instructions.

### 2.7 Microbiota analysis

Samples were prepared for high-throughput sequencing analysis of the entire DNA, and Illumina sequencing was performed as previously reported (14). The V3–V4 region of the 16S rRNA gene was chosen for PCR amplification by a forward primer (5’-ACTCCTACGGGAGGCAGCA-3’) and a reverse primer (5’-CGGACTACHVGGGTWTCTAAT-3’). The amplicon sequence variant (ASV)/operational taxonomic unit (OTU) was compared by quantitative analysis with the microbial ecology (QIIME2) platform, R software (version 3.2.0), and the Greengenes database. Then, α diversity, β diversity and different species screening were analyzed based on OTU/ASV and taxonomic ranks.

### 2.8 Analysis of faecal SCFAs

Mouse feces were weighed and added to sterile water at a ratio of 1:5 (W/V). They were mixed with vortex, extracted at 4°C for 12 h, and then centrifuged (12000 rpm/min, 10 min) at 4°C. Then, 200 μl of supernatant was absorbed before adding 40 μl of 25% (W/V) metaphosphate deproteinized 2-ethyl-butyric acid solution containing an internal standard to vortex mix and centrifuge at 4°C for 30 min in an ice bath (12000 rpm/min, 10 min). The supernatant was filtered through a 0.45 μm filter membrane to be detected. Different concentrations of acetic acid, propionic acid, isobutyric acid, butyric acid, isovaleric acid and valeric acid standard solutions were prepared.

The chromatographic column was DB-FFAP 30 m×0.32 mm×0.32 μm. Other conditions are as follows: carrier gas: high purity nitrogen; carrier gas flow: 2.2 mL/min; injection port: 250°C; detector: FID, 250°C; program heating conditions: the initial temperature of 60°C, with 10°C/min to 170°C and 8°C/min to 212°C.

### 2.9 Western blot analysis

A certain amount of fresh mouse colon tissue was weighed, and the protein cleavage buffer of protease inhibitor was added to the tissue homogenizer. Then, the homogenate was ground on ice, placed for 30 min on ice, and centrifuged (12000 rpm/min, 10 min) to take the supernatant for use. The protein concentration was detected by the BCA kit and quantitatively analyzed. Equal amounts of protein samples were separated by 10% SDS-PAGE electrophoresis and then transferred to the poly(vinylidene fluoride) (PVDF) membrane. The PVDF film was soaked in a sealing solution containing 5% skim milk powder, sealed in a shaker at room temperature for 2 h and incubated with primary antibody (1:500) at 4°C overnight. After adding the HPR-labeled secondary antibody for incubation at room temperature for 2 h, an ECL developer was added to develop the color in the automatic gel imaging system.

### 2.10 Bioinformatics and statistical analysis

QIIME2 (2019.4) software and R software (v3.2.0) were used for sequence data analysis. The obtained sequences were merged according to similarity, divided by ASV/OTU and studied by cluster analysis. According to the sparse curve of ASV/OTU clustering, the Chao1 index and Shannon index of α diversity index were analyzed and calculated to compare the richness and evenness among samples. The β diversity of microbiota from different groups was analyzed by NMDS based on the Bray-Curtis algorithm to describe the natural distribution characteristics among samples.

### 2.11 Statistical analysis

Statistical analysis was performed using GraphPad Prism software (version 5.01), and the significance of differences was analyzed using ANOVA (Tukey’s test). Significance was set at *P* < 0.05, and the results of test data were expressed as mean ± standard deviation.

## 3 Results

### 3.1 WQP relieved the signs and symptoms of UC in mice induced by DSS

**Figure 1:**
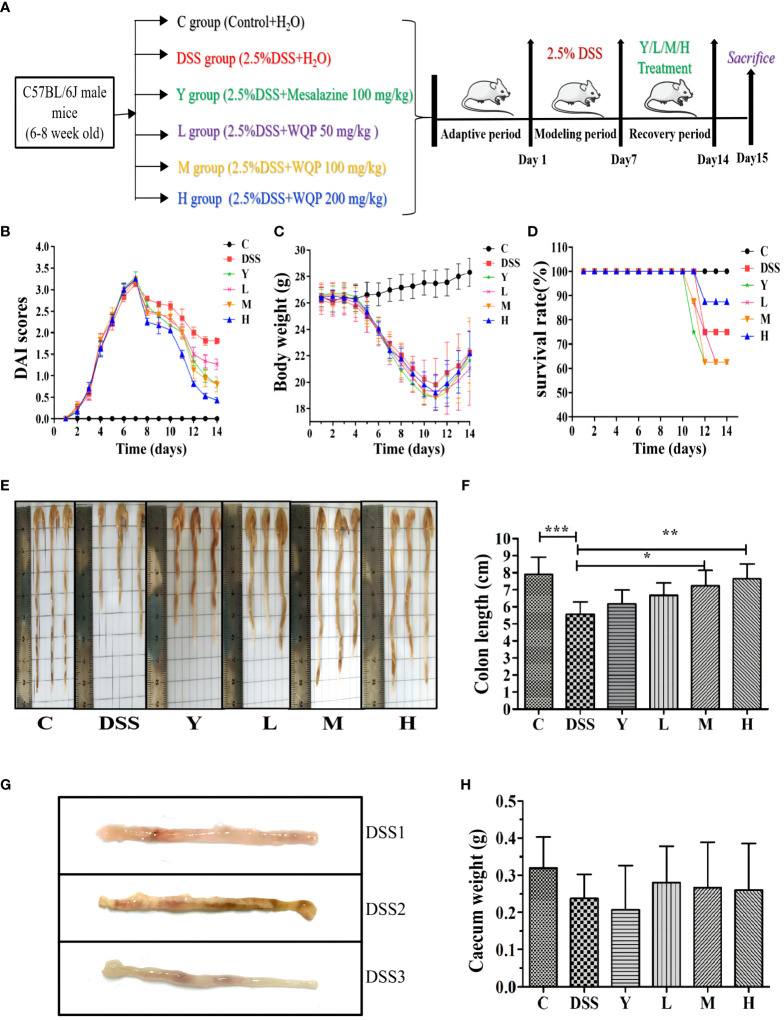
Observation of the general condition of mice. **(A)** A timeline of the experiment; **(B)** DAI scores; **(C)** Body weight; **(D)** Survival rate (%); **(E, F)** Colon length of the mice; **(G)** Bleeding points and superficial ulcers appears in the colon after the modeling period in the DSS group; **(H)** Caecum weight of the mice (g); C, normal control group; DSS, ulcerative colitis group; Y, positive control mesalazine group; L, low-dose WQP group; M, medium-dose WQP group; H, high-dose WQP group. Data are expressed as means ± SD (*n*= surviving mice/group). *P<0.05, **P<0.01, ***P<0.001.

The mice in the control group are agile, with normal weight gain and no hematochezia and diarrhea. As shown in **Figure 1**, the mice in other groups show obvious symptoms of colitis, mental malaise, slow response to external stimuli, messy coat without luster, weight loss, reduced diet, thin defecation and even hematochezia, death, obvious dirt, and swelling or even erosion around the anus. After 7 days of modeling, the DAI score of mice increased to 3, indicating the success of the UC model. After dissection, the colon was significantly shortened, with bleeding and edema, and was cut longitudinally. There were obvious bleeding points, superficial ulcers and other symptoms on the inner surface. **Figure 1G** indicates the success of the model. The survival rate of mice after modeling is shown in **Figure 1D**. Except for control group C, all mice in the other groups died during the recovery period of administration, 2 in the DSS group, 3 in Y, L and M groups, and 1 in the H group.

**Figure 1A** shows the model and administration scheme for this experiment. The mouse model was made for 7 days, and mice were administered for 7 days. The DAI index reflects the disease state of mice, and lower DAI values indicate better recovery effects. As shown in **Figure 1B**, the DAI values of groups M and H are lower after administration, indicating a good recovery effect. After intragastric administration of mesalazine and WQP, the above symptoms of mice in each group are improved in varying degrees, and the body weight of mice begins to increase slowly (as shown in **Figure 1C**). Body weight is an intuitive index to reflect the growth and health status of the body. Colon length is an indirect indicator of chronic inflammation and injury repair. As shown in **Figures 1E, F**, the colon in the DSS group is significantly shorter than that in group C (*P* < 0.001). The colon length of the mesalazine group and WQP group recovers compared with that of the DSS group. There is a significant difference between M and H groups (*P* < 0.05), with a good recovery effect. As shown in **Figure 1H**, the cecal weight is larger in L, M and H groups than in the Y group, but there is no significant difference. After WQP administration, the cecal weight of mice is close to that of the normal group, but there is no significant difference.

### 3.2 Pathological structure analysis of mouse colonic tissue

**Figure 2:**
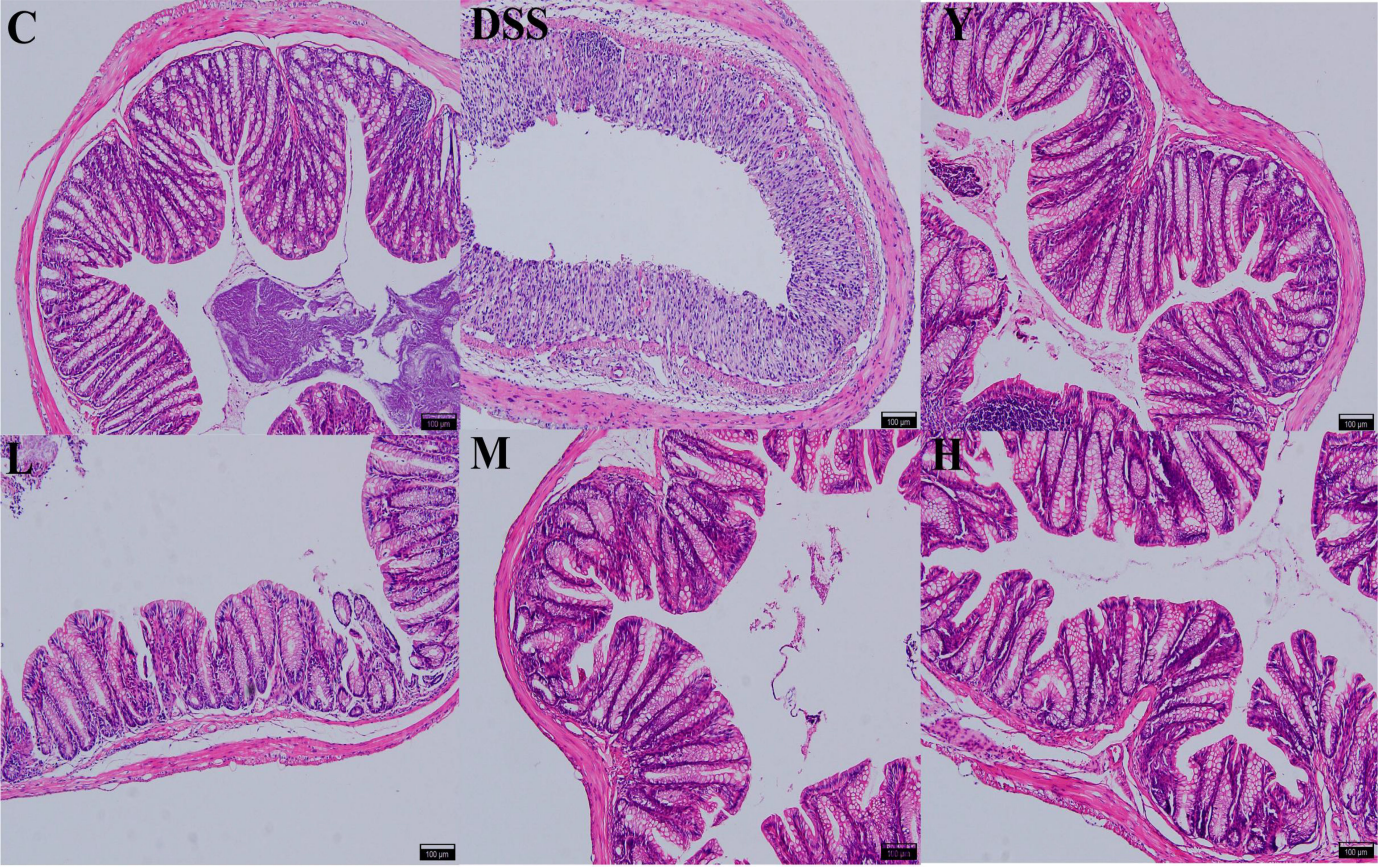
Histopathological observation of the colon (HE, 100×). C, normal control group; DSS, ulcerative colitis group; Y, positive control mesalazine group; L, low-dose WQP group; M, medium-dose WQP group; H, high-dose WQP group.

As shown in **Figure 2**, HE staining shows that the structure of the colon in normal control group C is clear, with neatly arranged mucosal epithelial cells, no villus necrosis and exfoliation, and abundant goblet cells. In the DSS group, normal saline was used for natural recovery for 7 days after modeling. The intestinal wall of the colon was microscopically found to be intact, but inflammatory cells were infiltrated, with decreased or even disappeared glands, atrophied and sparse intestinal villi, and destroyed crypts. Compared with the case in the DSS group, the colonic tissue of mice fed with mesalazine and WQP approaches normal. The intestinal villi of the M and H groups are longer, the crypt is deeper, and the recovery effect is better than those of the L group. Therefore, WQP can improve the colon injury caused by DSS and restore the intestinal villus structure, thus protecting the intestinal mucosa, especially in group H.

### 3.3 Determination of cytokines in colon tissue

**Figure 3:**
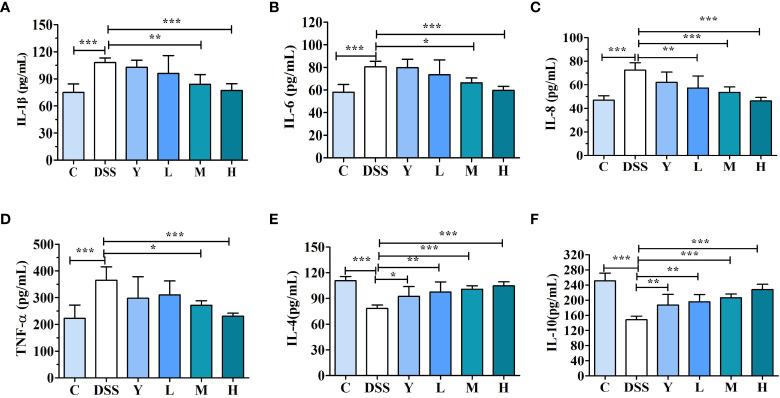
Cytokine content in mouse colonic tissue. **(A)** IL-1β, **(B)** IL-6, **(C)** IL-8, **(D)** TNF-α, **(E)** IL-4, and **(F)** IL-10 levels in the mouse colonic tissue were analyzed by ELISA kits. C, normal control group; DSS, ulcerative colitis group; Y, positive control mesalazine group; L, low-dose WQP group; M, medium-dose WQP group; H, high-dose WQP group. Data are expressed as means±SD (*n*=6). *P<0.05, **P<0.01, ***P<0.001.

As shown in **Figure 3**, the contents of cytokines IL-1β, IL-6, IL-8, TNF-α, IL-4 and IL-10 in mouse colons are determined. The effects of WQP on colonic inflammation can be reflected by measuring the contents of cytokines. The contents of pro-inflammatory cytokines IL-1β, IL-6, IL-8 and TNF-α in the DSS group are significantly higher than those in group C (*P* < 0.001), which may be due to an increase of pro-inflammatory cytokines caused by the imbalance of gut microbiota. The contents of IL-1β, IL-6, IL-8 and TNF-α in the M and H groups decrease significantly (*P* < 0.05) compared with those in the DSS group, and the content of cytokines in the L group also decreases, but there is no significant difference. The contents of anti-inflammatory cytokines IL-4 and IL-10 in the DSS group are significantly lower than those in group C (*P* < 0.001). The contents of IL-4 and IL-10 in the Y, L, M and H groups are significantly higher (*P* < 0.05) compared with those in the DSS group, and the level of inflammatory cytokines in the H group is closer to that in the C group. These results show that WQP can significantly improve the colitis injury caused by DSS.

### 3.4 Effects of WQP on the diversity and structural composition of gut microbiota in mice feces

#### 3.4.1 Analysis of α and β diversity

**Figure 4:**
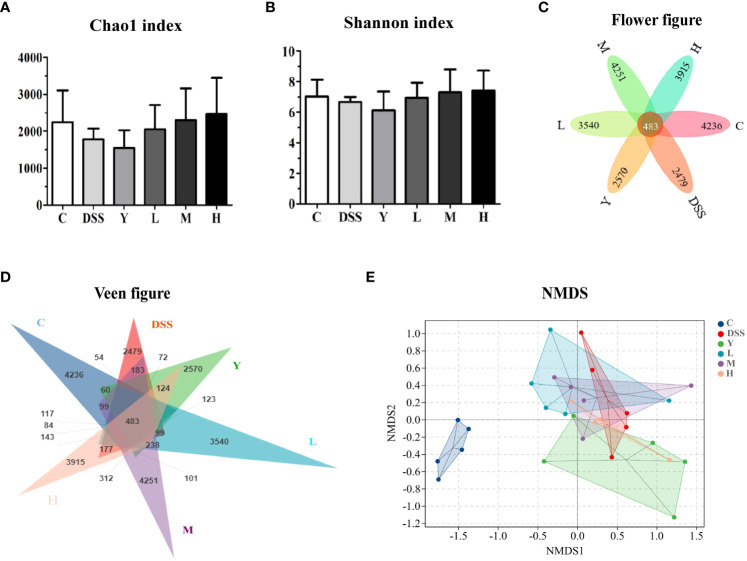
Analysis of α and β diversity indices. **(A)** Chao1 index; **(B)** Shannon index; **(C)** Flower figure; **(D)** Veen figure; **(E)** NMDS analysis. C, normal control group; DSS, ulcerative colitis group; Y, positive control mesalazine group; L, low-dose WQP group; M, medium-dose WQP group; H, high-dose WQP group. Data are expressed as means ± SD (*n*=5).

The composition and diversity of gut microbiota in the fresh feces of mice were determined. The results of α and β diversity analysis are shown in **Figure 4**. Among them, α diversity can indicate the species diversity within a single sample. Higher values of the Chao1 index in the α diversity index suggest higher community richness. The Shannon diversity index comprehensively considers the richness and evenness of the community, and higher index values indicate higher community diversity. In this study, QIIME software was used to analyze the α diversity (Chao1 richness index and Shannon diversity index) of gut microbiota in different treatment groups. The results are shown in **Figures 4A, B**. As shown in **Figure 4A**, the Chao1 diversity index of the DSS group is lower than that of normal control group C, and the Chao1 diversity index of the L, M and H groups is higher than that of the DSS group; the diversity index of the H group is the highest, but there is no significant difference (*P* > 0.05). As shown in **Figure 4B**, the Shannon diversity index shows no significant difference among different groups (*P* > 0.05) but is higher in L, M and H groups than in the DSS group. The Flower figure and Veen figure in **Figures 4C, D** show that there are 483 OTUs among the six groups, and the number of OTUs in the DSS group is the least. The number of OTUs in the L, M and H groups is close to that in normal control group C. The results show that the diversity of gut microbiota decreases significantly after the establishment of the DSS model, and WQP can inhibit the reduction in the OTU of gut microbiota caused by DSS and retain part of gut microbiota. The non-metric multidimensional scaling (NMDS) analysis shown in **Figure 4E** can describe the characteristics of natural distribution among samples. Since NMDS adopts hierarchical ranking, it can be approximately considered that the closer the distance between two points, the smaller the difference in microbial communities between the two samples. **Figure 4E** shows that the points of the representative samples in normal control group C and group DSS are significantly separated, indicating that the structure of the gut microbiota of mice changes significantly after modeling. Most of the representative points of the L, M and H groups are gathered together, indicating a similar composition of the microbiota. The results show that WQP can increase the richness and diversity of gut microbiota in UC model mice.

#### 3.4.2 Structural composition analysis of gut microbiota

**Figure 5:**
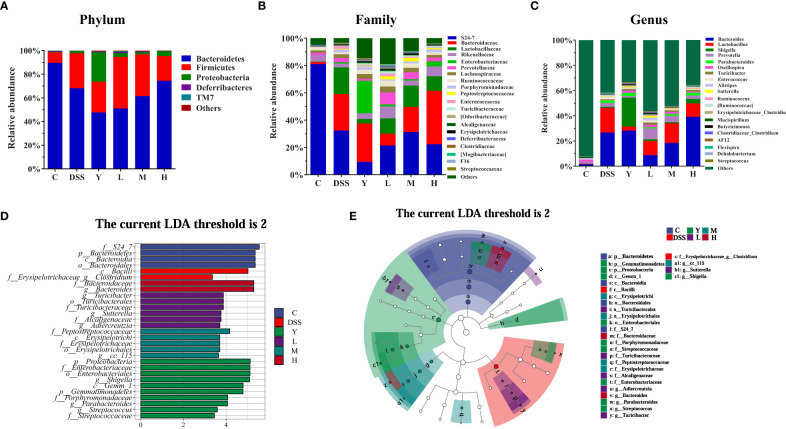
Composition analysis of the gut microbiota. **(A)** Phylum level; **(B)** Family level; **(C)** Genus level; **(D)** LDA score distribution histogram; **(E)** Evolutionary cladogram. The threshold of the score of LDA analysis is 2; C, normal control group; DSS, ulcerative colitis group; Y, positive control mesalazine group; L, low-dose WQP group; M, medium-dose WQP group; H, high-dose WQP group. Data are expressed as means ± SD (*n*=5).

After 16S rRNA sequencing, the changes in the structure and relative abundance of fecal gut microbiota can be more clearly obtained by analyzing mice gut microbiota at the phylum, family and genus levels according to the results of OTU division and taxonomic status identification. As shown in **Figure 5A**, the relative abundance of gut microbiota in each group of samples is shown at the phylum level. The results show that the fecal gut microbiota of mice is mainly composed of *Bacteroidetes*, *Firmicutes*, *Proteobacteria*, *Deferribacteres* and *TM7*. The relative abundance of *Bacteroidetes*, *Deferribacteres* and *TM7* decreases, and that of *Firmicutes* and *Proteobacteria* increases in the DSS group compared with those in group C. The relative abundance of *Bacteroidetes* and *Firmicutes* in the Y group decreases, while that of *Proteobacteria*, *Deferribacteres* and *TM7* increases compared with those in the DSS group. The relative abundance of *Bacteroidetes* decreases, and that of *Firmicutes* increases in group L. The relative abundance of *Bacteroidetes* increases, and that of *Firmicutes* decreases in group H, which is closer to the case in group C. **Figure 5B** shows the relative abundance of gut microbiota at the family level. At the family level, the gut microbiota of mice is mainly composed of *S24-7*, *Bacteroidaceae*, *Lactobacillaceae, Rikenellaceae* and so on. Compared with group C, all the other groups show a significantly decreased relative abundance of *S24-7* and a significantly increased relative abundance of *Bacteroidaceae* after modeling. The relative abundance of *Rikenellaceae* in groups Y, L, M and H increases significantly compared with that in the DSS group, which approaches the case in group C. **Figure 5C** shows the change in the relative abundance of gut microbiota at the genus level. At the genus level, the gut microbiota of mice is mainly composed of *Bacteroides*, *Lactobacillus*, *Shigella*, *Prevotella*, *Parabacteroides* and *Oscillospira*. Compared with group C, the other groups show a significantly increased relative abundance of *Bacteroides*, *Lactobacillus*, *Shigella* and *Parabacteroides*. The relative abundance of *Bacteroides*, *Lactobacillus* and *Parabacteroides* in groups L and M decreases significantly, while that of *Prevotella* and *Oscillospira* increases significantly compared with those in the DSS group. In group H, the relative abundance of *Bacteroides*, *Shigella* and *Oscillospira* increases significantly, while that of *Lactobacillus* and *Prevotella* decreases significantly. In group Y, the relative abundance of *Bacteroides*, *Shigella* and *Oscillospira* increases significantly, while that of *Lactobacillus* and *Prevotella* decreases significantly.

**Figures 5D, E** show the fecal microbial taxa determined by LEfSe analysis, which vary most among all groups. In the LEfSe method, the LDA score 2 was used as the screening criterion to determine the microorganisms with high abundance in each group. As shown, at the family and genus level, the highest significance is *f_S24_7* in group C; *f_Erysipelotrichaceae_g_Clostridium* in group DSS; *g_Shigella* in group Y; *g_Turicibacter* in group L; *f_Peptostreptococcaceae* in group M; *g_Bacteroides* in group H. These results suggest that different doses of WQP can regulate the structure of gut microbiota and accelerate the recovery of gut microbiota in UC mice model.

### 3.5 Analysis of faecal SCFAs

**Figure 6:**
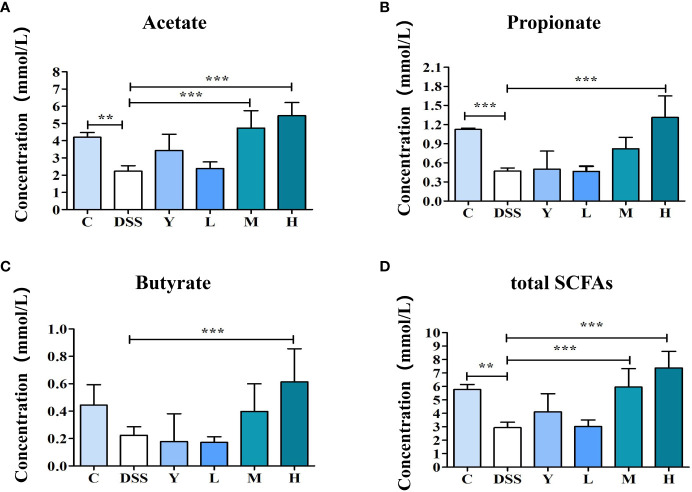
The content of SCFAs in fresh feces of mice. **(A)** Acetate concentration, **(B)** Propionate concentration, **(C)** Butyrate concentration, and **(D)** total SCFAs levels in fresh feces of mice. C, normal control group; DSS, ulcerative colitis group; Y, positive control mesalazine group; L, low-dose WQP group; M, medium-dose WQP group; H, high-dose WQP group. Data are expressed as means±SD (n=5). **P<0.01, ***P<0.001.

The SCFAs in mouse feces are mainly composed of acetate, propionate and butyrate, among which acetate is the most abundant. As shown in **Figure 6**, the contents of acetate, propionate and total SCFAs in the DSS group are significantly lower than those in group C (*P* < 0.01), and the content of butyrate is also lower than that in group C, but the difference is not significant. The contents of acetate and total SCFAs in the M group are significantly higher (*P* < 0.001) than those in the DSS group, and the contents of propionate and butyrate also increase, but the difference is not significant. The contents of acetate, propionate, butyrate and total SCFAs in the H group are significantly higher than those in the DSS group (*P* < 0.001). Moreover, SCFAs can provide energy for intestinal epithelial cells, reduce intestinal inflammation and participate in immunity. High doses of WQP can significantly increase the content of SCFAs.

### 3.6 Effects of WQP on the expression of tight junction proteins

**Figure 7:**
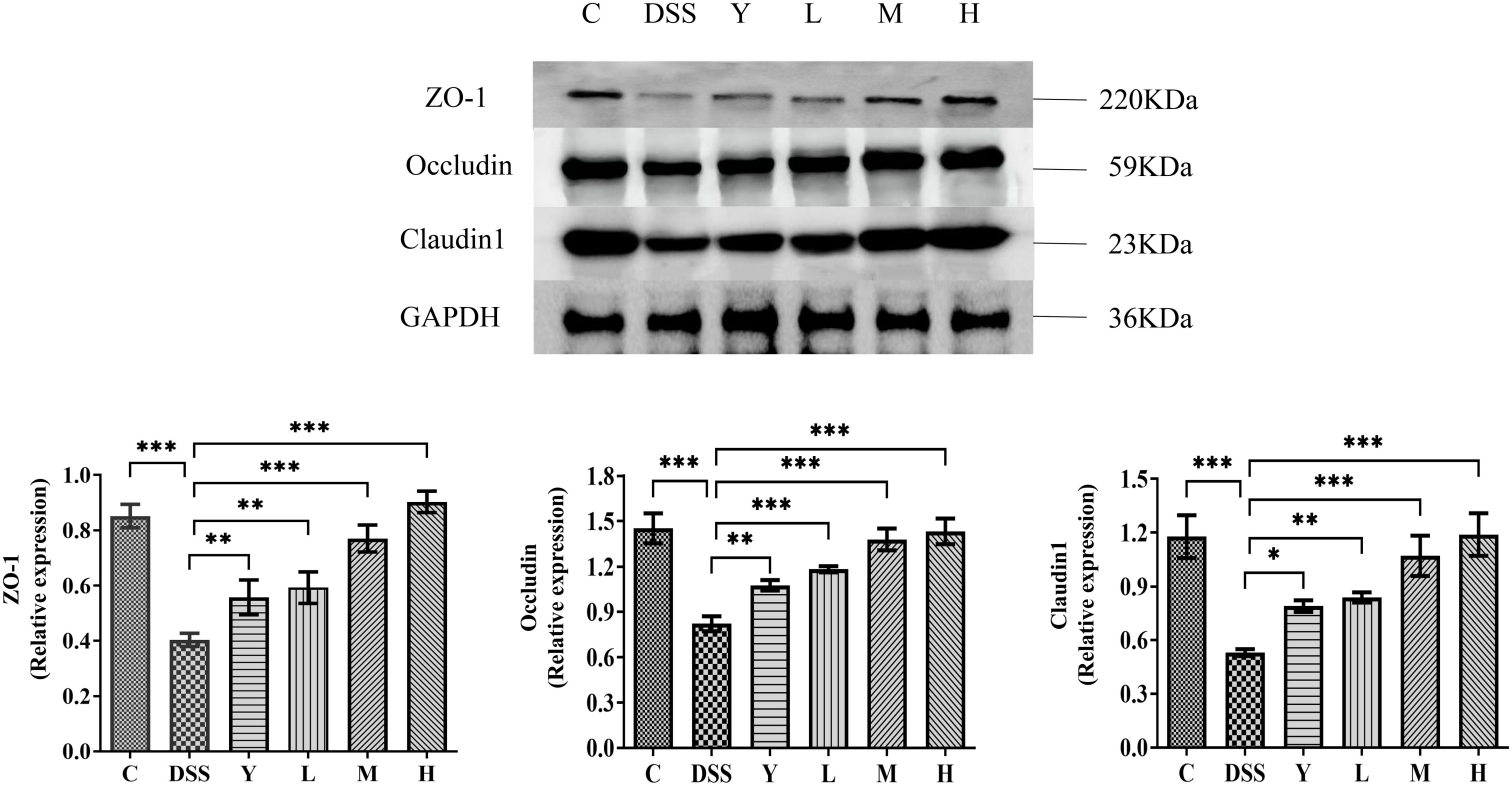
The expression of tight junction proteins in mouse colon tissue. C, normal control group; DSS, ulcerative colitis group; Y, positive control mesalazine group; L, low-dose WQP group; M, medium-dose WQP group; H, high-dose WQP group. Data are expressed as means ± SD (*n*=5). *P<0.05, **P<0.01, ***P<0.001.

The expression of tight junction proteins ZO-1, Occludin and Claudin-1 in mouse colon tissue is measured and shown in **Figure 7**. The expression of tight junction proteins ZO-1, Occludin and Claudin-1 in the DSS group is significantly lower than that in group C (*P* < 0.001). The expression of tight junction proteins ZO-1, Occludin and Claudin-1 in the Y, L, M and H groups is significantly increased (*P* < 0.05) compared with that in the DSS group. This result shows that different doses of WQP can protect the intestinal barrier by increasing the expression level of tight junction proteins.

### 3.7 Correlation of gut microbiota with acute colitis-related indexes

**Figure 8:**
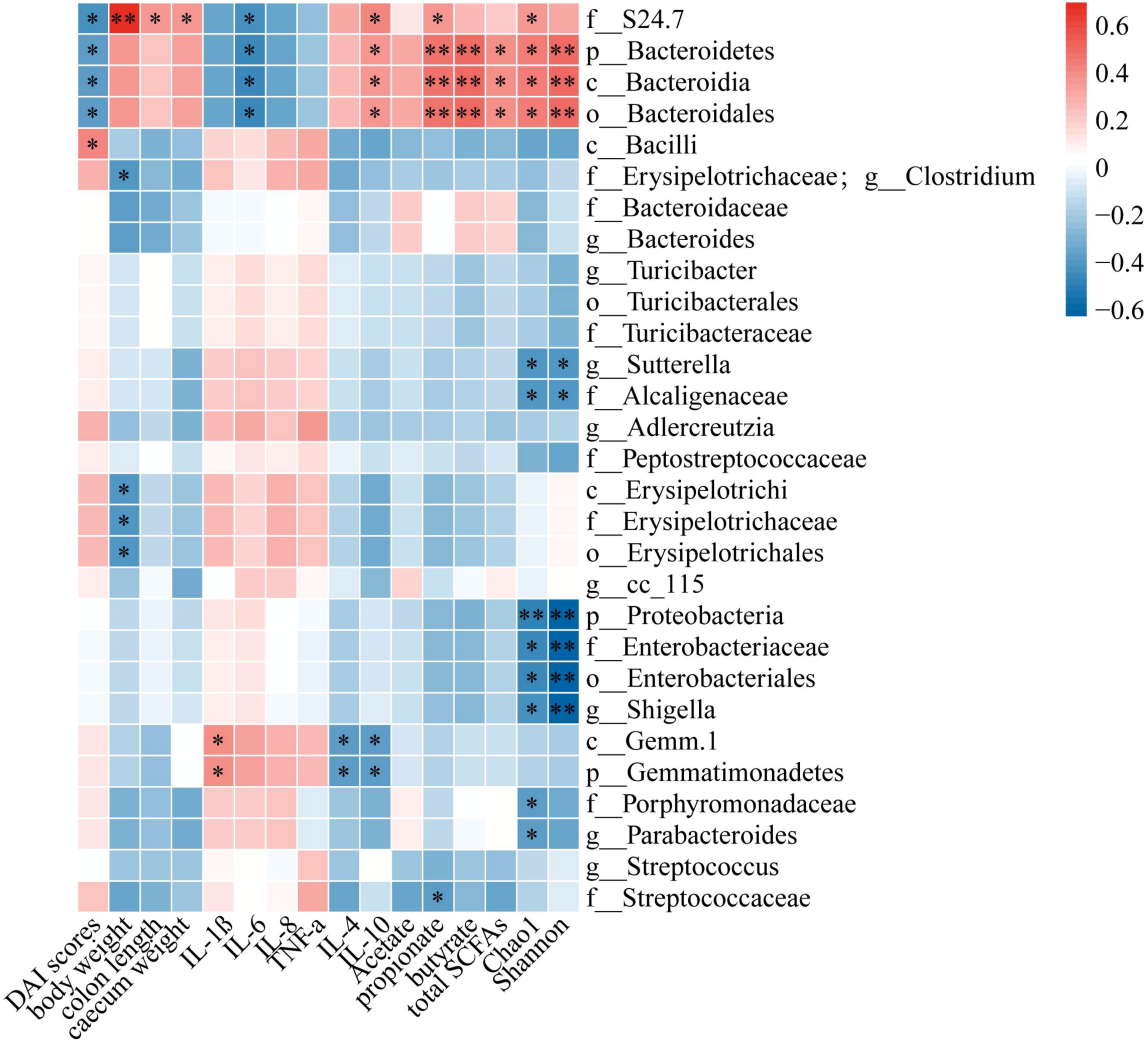
Heatmap showed Pearson’s rank correlation between the relative abundance of gut microbiota and DSS-induced colitis-related indices of mice. Red color: positive correlation; white color: no correlation; and blue color: negative correlation. Signifificant difffference was indicated by asterisks.*P < 0.05, **P < 0.01.

To investigate the possible association between gut microbiota and DSS-induced acute colitis-related indexes, the data were analyzed using the Pearson’s rank correlation. As shown in **Figure 8**, the DAI scores were positively correlated with *c_Bacilli*, *f_Erysipelotrichaceae;g_Clostridium*, *g_Adlercreutzia*, *c_Erysipelotrichi*, *f_Erysipelotrichaceae*, *o_Erysipelotrichales* and were negatively correlated with f*_S24-7*, *p_Bacteroidetes*, *c_Bacteroidia* and *o_Bacteroidales*. Among these indexes, the body weight, colon length, caecum weight, IL-4, IL-10, acetate, propionate, butyrate, total SCFAs, Chao1 and Shannon were positively correlated with *f_S24-7*, *p_Bacteroidetes*, *c_Bacteroidia*, *o_Bacteroidales.* Meanwhile, the expressions of inflammatory cytokines (IL-1β, IL-6, IL-8 and TNF-α) were positively correlated with *c_Gemm-1*, *p_Gemmatimonadetes* and negatively correlated with *f_S24-7*, *p_Bacteroidetes*, *c_Bacteroidia*, and *o_Bacteroidales*. Besides, the Chao1 and Shannon were negatively correlated with *g_Sutterella*, *f_Alcaligenaceae*, *p_Proteobacteria*, *f_Enterobacteriaceae*, *o_Enterobacteriales*, *g_Shigella* and so on.

## 4 Discussion

The pathogenesis of UC, which is a chronic inflammatory disease of the colon, involves many factors, including genetic susceptibility, epithelial barrier defects, immune response disorders, environmental factors, diet, and antibiotics. Its pathogenesis is still unclear and mainly due to the interaction of multiple factors, such as intestinal mucosal barrier deficiency, intestinal immune dysfunction, and intestinal microbial imbalance (17). The DSS-induced UC model is currently the best animal disease model consistent with the pathological characteristics of human UC, often manifesting as weight loss, hemorrhagic diarrhea, bloody stool, colon shortening, and histological and pathological changes in the colon (18). In addition, the model has the advantages of simple establishment, low cost and short time. Therefore, in this study, the DSS-induced colitis model was used to investigate the efficacy of WQP in UC mice. The results showed that after continuous administration of 2.5% DSS drinking water for 7 days, C57BL/6J mice showed various physiological and pathological manifestations of UC, including bloody, sparse stool or watery stool, weight loss, colon shortening, and histopathological changes of the colon. After administration of WQP, compared with the DSS group, L, M and H groups showed improved diarrhea state, decreased DAI index, and improved colonic tissue length and pathological changes, with the H group exhibiting better effects. Thus, WQP can significantly reduce the content of pro-inflammatory cytokines and increase the content of anti-inflammatory cytokines in colon tissue; increase the expression of tight junction proteins and protect intestinal mucosal barrier; increase the richness and diversity of gut microbiota and regulate the structure and relative abundance of gut microbiota at the phylum, family and genus levels; proliferate beneficial bacteria and inhibit harmful bacteria; increase the contents of gut microbiota metabolite SCFAs and exert an anti-UC effect.

As an indicator of disease severity, the DAI score can reflect the pathological state of mice. In addition, colon shortening, generally considered a sign of inflammation, was also observed in the DSS group. Compared with the DSS group, different doses of WQP improved the body weight and colon length of mice and reduced the DAI score, with higher doses showing better results. In addition, HE staining showed that DSS destroyed the villous structure of the colon and caused inflammatory cell infiltration and crypt destruction. In contrast, administration of WQP significantly reduced mucosal inflammatory cell infiltration and increased the length of intestinal villi and crypt depth, with better recovery in group H. These results suggest that WQP can improve DSS-induced colitis in mice.

Pro-inflammatory cytokines such as IL-1β, IL-6, IL-8 and TNF-α can exacerbate UC. The administration of WQP significantly decreased the expressions of these inflammatory factors in the colon. The level of inflammatory factors can be used to indicate the inflammation degree of the intestinal mucosa. A study has found that in the mouse colitis model induced by DSS, intragastric administration of taxifolin can decrease the levels of inflammatory factors TNF-α, IL-6 and IL-1β, increase the secretion of anti-inflammatory factors IL-10, superoxide dismutase and immunoglobulin, inhibit NF-κB signaling, and restore the composition and function of gut microbiota by increasing the expression of tight junction proteins, thereby significantly alleviating the symptoms and histological changes of colitis (19).

Various vegetable drug polysaccharides have been reported to regulate gut microbiota under different conditions and play a key role in chronic diseases (20). *Scutellaria baicalensis Georgi* polysaccharides can inhibit the production of pro-inflammatory cytokines IL-1β, IL-6, and TNF-α and improve DSS-induced UC by improving intestinal barrier function and regulating intestinal microorganisms (21). *Crataegus pinnatifida* polysaccharides attenuate DSS-induced UC in mice by regulating gut microbiota composition, inhibiting the expression of inflammatory cytokines IL-6, IL-1β, and TNF-α and increasing the content of SCFAs (22). *Ganoderma lucidum* polysaccharides can alleviate DSS-induced UC in mice by regulating disordered intestinal microorganisms, inhibiting the TLR4/MyD88/NF-κB signaling pathway, increasing the content of SCFAs and improving intestinal barrier function (23). *Panax ginseng* polysaccharides and their effective subfractions can improve DSS-induced colitis in mice by decreasing the expression of colitis-related proteins IL-1β, IL-2, IL-6 and IL-17, up-regulating the expression of tight junction-related proteins ZO-1 and Occludin, regulating the diversity and composition of gut microbiota, increasing the production of SCFAs and inhibiting the TLR4/MYD88/NF-κB signaling pathway (12). Therefore, many polysaccharides can play a positive role in regulating gut microbiota. In this paper, WQP can improve DSS by regulating the structure and diversity of gut microbiota in mice at the phylum, family and genus levels. Specifically, high-dose WQP can regulate the composition of the gut microbiota of DSS by increasing the relative abundance of *Bacteroidetes*, *Rikenellaceae*, *Shigella*, and *Oscillospira*.

Gut microbiota can affect the health of the host by producing metabolites. As metabolites of gut microbiota, SCFAs are mainly produced by anaerobic hydrolysis of dietary fibers in the intestine, such as oligosaccharides and inulin, in which acetic acid, propionic acid and butyric acid are the most abundant metabolites (24). The butyric acid produced by fermentation is mainly absorbed by colonic epithelial cells, propionic acid is immediately absorbed by the liver through the portal vein, and acetic acid is mainly metabolized in various tissues to perform its physiological functions (25). Butyric acid-producing bacteria in the intestinal tract are mainly Gram-positive thick-walled bacteria, propionic acid-producing bacteria mainly belong to *Bacteroidetes*, and acetic acid-producing bacteria are more extensive, mainly *Bifidobacterium* and *Bacteroides*. Moreover, the imbalance of gut microbiota can inhibit the transformation of SCFAs, change the intestinal environment and aggravate the disorder of gut microbiota, thus inducing disease. SCFAs are the main energy source of intestinal epithelial cells and can maintain the acid-base balance of the intestinal tract and inhibit the survival of harmful bacteria. They are important metabolites in intestinal microbial environments and closely related to immune, anti-inflammatory and anti-tumor activities (26). Changes in the metabolites of microflora can reflect the microecological homeostasis of the intestinal tract. A study has shown that SCFAs, as the substrate of energy metabolism of intestinal epithelial cells, participate in the functional integrity of intestinal epithelial cells and regulate immune function, with acetic acid being the main fermentation metabolite of most bacteria and an important energy source of cells (27). Propionic acid absorbed through the colon can serve as a main source of energy for the liver and inhibit the synthesis of cholesterol in the liver. Different carbohydrate structures are fermented by gut microbiota to produce different SCFAs. In this study, WQP ameliorated the decrease of SCFAs content caused by DSS and promoted the increased content of acetic acid, propionic acid, butyric acid and total SCFAs, with the H group showing a more significant effect. In general, high-dose WQP is more effective in improving the inflammatory symptoms of UC induced by DSS.

The intestinal barrier mainly includes the immune barrier, mechanical barrier, microbial barrier and chemical barrier, which have a corresponding structural basis to prevent harmful intestinal substances and pathogens from entering the body environment and maintain its stability. Among them, the intestinal mucosal mechanical barrier comprises intestinal mucosal epithelial cells and intercellular tight junctions. The tight junctions are mainly composed of a series of membrane-penetrating proteins and cytoplasmic proteins, including occlusal proteins (occludin), closure proteins (claudin), banded closure proteins (zonula occludens, zo) families, and junction adhesion molecules (junctional adhesion molecule) (28). The expression of intercellular tight junction proteins is related to UC, whose core pathological feature is the loss of intestinal barrier integrity, which leads to increased bacterial antigen translocation, thus stimulating the inflammatory response of intestinal mucosa (29). Tight junction proteins play a decisive role in maintaining intestinal mucosal barrier function and are important components in maintaining the structural integrity of the intestinal mucosal barrier. Once tight junctions are lost or mutated, intercellular permeability will be significantly increased, and intestinal barrier function will be impaired (30). Therefore, tight junction proteins play an important role in maintaining the integrity of the intestinal barrier.

Impaired intestinal mucosal barrier function is considered to be a direct cause of UC (31). As the tissue structure with the largest contact area between the body and the external environment, the intestinal mucosa can prevent the invasion of pathogens, antigens and toxins. Common indicators used to evaluate the integrity of intestinal mucosal function are the structural integrity of intestinal tissue, the expression of mucosal proteins, the level of pro-inflammatory cytokines and intestinal bacterial translocation (32). Many experimental colitis experiments have shown that the development of intestinal inflammation is related to the defect of the intestinal mucosal barrier. The intestinal barrier structure is mainly composed of tight junction monolayer intestinal epithelial cells, in which tight junction proteins ZO-1, Occludin and Claudin-1 play a major role. In this study, the protein expression of ZO-1, Occludin and Claudin-1 was significantly down-regulated in the DSS group, suggesting that DSS destroyed the mucous layer and increased intestinal permeability. Additionally, different doses of WQP significantly increased the expression of tight junction proteins, which play a protective role in the intestinal barrier. Therefore, WQP may have a beneficial effect on UC by improving the integrity of the intestinal mucosal barrier.

This paper discussed the ameliorative effect of WQP on UC mice. However, its mechanism needs to be further studied. In the next work, we will deeply study the specific mechanism of WQP in improving and treating UC to provide a data basis for clinical research.

## 5 Conclusions

WQP can improve the disease state of UC mice. Compared with the DSS group, L, M and H WQP groups showed a significantly reduced DAI index, with a significantly increased colon length in M and H groups. Additionally, L, M, and H WQP groups reduced colonic inflammatory infiltration and tissue structure damage, with a better effect in the H group than in the M and L groups. Group M and H showed a significantly decreased content of pro-inflammatory cytokines IL-1β, IL-6, IL-8 and TNF-α, significantly increased content of IL-4 and IL-10, alleviated colitis symptoms, increased expression levels of tight junction proteins ZO-1, Occludin and Claudin-1, and protected intestinal mucosal barrier. High doses of WQP increased the relative abundance of *Bacteroidetes*, *Rikenellaceae*, *Bacteroides*, *Shigella* and *Oscillospira* and reduced that of *Lactobacillus* and *Prevotella*, making the fecal microbiota structure of mice close to that in the normal control group. Moreover, WQP can increase the content of faecal SCFAs, the metabolite of flora, and reduce intestinal inflammation. The results of this study can provide a data basis for the improvement and therapeutic effect of natural polysaccharides on colitis and promote the development and application of functional products of *Panax quinquefolius* and its active components.

## Data availability statement

The datasets presented in this study can be found in online repositories. The names of the repository/repositories and accession number(s) can be found below: PRJNA934750 (SRA).

## Ethics statement

The animal care and experimental procedures used in this study were approved by the Laboratory Animal Management and Ethics Committee of the Institute of Special Animal and Plant Sciences, Chinese Academy.

## Author contributions

D-DR: Methodology, data curation, writing-original draft, writing-reviewing and editing. K-CC: Conceptualization, methodology, formal analysis. S-SL: Supervision, validation, writing-reviewing and editing. Y-TZ: Visualization, software. Z-ML: Investigation. SL: Supervision. Y-SS: Project administration, writing-reviewing and editing. All authors contributed to the article and approved the submitted version.

## References

[1] Hodson R. Inflammatory bowel disease. Nature (2016) 540(7634):S97. doi: 10.1038/540S97a 28002398

[2] Levine A Rhodes JM Lindsay JO Abreu MT Kamm MA Gibson PR. Dietary guidance from the international organization for the study of inflammatory bowel diseases. Clin Gastroenterol hepatol: Off Clin Pract J Am Gastroenterol Assoc (2020) 18(6):1381–92. doi: 10.1016/j.cgh.2020.01.046 32068150

[3] Gajendran M Loganathan P Jimenez G Catinella AP Ng N Umapathy C. A comprehensive review and update on ulcerative colitis. Disease-A-Month: DM (2019) 65(12):100851. doi: 10.1016/j.disamonth.2019.02.004 30837080

[4] Li C Wang J Ma R Li L Wu W Cai D. Natural-derived alkaloids exhibit great potential in the treatment of ulcerative colitis. Pharmacol Res (2022) 175:105972. doi: 10.1016/j.phrs.2021.105972 34758401

[5] Zhang XJ Yuan ZW Qu C Yu XT Huang T Chen PV. Palmatine ameliorated murine colitis by suppressing tryptophan metabolism and regulating gut microbiota. Pharmacol Res (2018) 137:34–46. doi: 10.1016/j.phrs.2018.09.010 30243842

[6] Matijašić M Meštrović T Perić M Čipčić Paljetak H Panek M Vranešić Bender D. Modulating composition and metabolic activity of the gut microbiota in IBD patients. Int J Mol Sci (2016) 17(4):578. doi: 10.3390/ijms17040578 27104515 PMC4849034

[7] Rooks MG Garrett WS. Gut microbiota, metabolites and host immunity. Nat Rev Immunol (2016) 16(6):341–52. doi: 10.1038/nri.2016.42 PMC5541232 27231050

[8] Okumura R Takeda K. Maintenance of intestinal homeostasis by mucosal barriers. Inflammation And Regeneration (2018) 38:5. doi: 10.1186/s41232-018-0063-z 29619131 PMC5879757

[9] Xavier RJ Podolsky DK. Unravelling the pathogenesis of inflammatory bowel disease. Nature (2007) 448(7152):427–34. doi: 10.1038/nature06005 17653185

[10] Wilhelm SM Mckenney KA Rivait KN Kale-Pradhan PB. A review of infliximab use in ulcerative colitis. Clin Ther (2008) 30(2):223–30. doi: 10.1016/j.clinthera.2008.02.014 18343261

[11] Creed TJ Probert CS Norman MN Moorghen M Shepherd NA Hearing SD. Basiliximab for the treatment of steroid-resistant ulcerative colitis: further experience in moderate and severe disease. Alimentary Pharmacol Ther (2006) 23(10):1435–42. doi: 10.1111/j.1365-2036.2006.02904.x 16669958

[12] Li S Huo X Qi Y Ren D Li Z Qu D. The protective effects of ginseng polysaccharides and their effective subfraction against dextran sodium sulfate-induced colitis. Foods (2022) 11(6):890. doi: 10.3390/foods11060890 35327312 PMC8949837

[13] Guo M Shao S Wang D Zhao D Wang M. Recent progress in polysaccharides from *panax ginseng* c. A. Meyer. Food Funct (2021) 12(2):494–518. doi: 10.1039/d0fo01896a 33331377

[14] Ren DD Li SS Lin HM Xia YS Li ZM Bo PP. *Panax quinquefolius* polysaccharides ameliorate antibiotic-associated diarrhoea induced by lincomycin hydrochloride in rats via the MAPK signaling pathways. J Immunol Res (2022) 2022:4126273. doi: 10.1155/2022/4126273 35345778 PMC8957475

[15] Ghosh R Smith SA Nwangwa EE Arivett BA Bryant DL Fuller ML. *Panax quinquefolius* (north american ginseng) cell suspension culture as a source of bioactive polysaccharides: immunostimulatory activity and characterization of a neutral polysaccharide AGC. Int J Biol Macromol (2019) 139:221–32. doi: 10.1016/j.ijbiomac.2019.07.215 31376448

[16] Ashour RH Hazem NM AbdElfattah AA El-Kady RA Elmasry A. Pentosan polysulfate sodium augments the therapeutic effect of 5-aminosalicylic acid in DSS colitis model; the role of IL-35 expression. Int immunopharmacol (2022) 106:108620. doi: 10.1016/j.intimp.2022.108620 35247859

[17] Ungaro R Mehandru S Allen PB Peyrin-Biroulet L Colombel JF. Ulcerative colitis. Lancet (2017) 389(10080):1756–70. doi: 10.1016/S0140-6736(16)32126-2 PMC6487890 27914657

[18] Das S Batra SK Rachagani S. Mouse model of dextran sodium sulfate (dss)-induced colitis. Bio-Protocol (2017) 7(16):e2515. doi: 10.21769/BioProtoc.2515 34541176 PMC8413514

[19] Hou J Hu M Zhang L Gao Y Ma L Xu Q. Dietary taxifolin protects against dextran sulfate sodium-induced colitis via NF-κB signaling, enhancing intestinal barrier and modulating gut microbiota. Front Immunol (2021) 11:631809. doi: 10.3389/fimmu.2020.631809 33664740 PMC7921741

[20] Zhang T Yang Y Liang Y Jiao X Zhao C. Beneficial effect of intestinal fermentation of natural polysaccharides. Nutrients (2018) 10(8):1055. doi: 10.3390/nu10081055 30096921 PMC6116026

[21] Cui L Guan X Ding W Luo Y Wang W Bu W. *Scutellaria baicalensis* georgi polysaccharide ameliorates dss-induced ulcerative colitis by improving intestinal barrier function and modulating gut microbiota. Int J Biol Macromol (2021) 166:1035–45. doi: 10.1016/j.ijbiomac.2020.10.259 33157130

[22] Guo C Wang Y Zhang S Zhang X Du Z Li M. *Crataegus pinnatifida* polysaccharide alleviates colitis via modulation of gut microbiota and SCFAs metabolism. Int J Biol Macromol (2021) 181:357–68. doi: 10.1016/j.ijbiomac.2021.03.137 33774071

[23] Guo C Guo D Fang L Sang T Wu J Guo C. *Ganoderma lucidum* polysaccharide modulates gut microbiota and immune cell function to inhibit inflammation and tumorigenesis in colon. Carbohydr Polymers (2021) 267:118231. doi: 10.1016/j.carbpol.2021.118231 34119183

[24] Xu C Marques FZ. How dietary fibre, acting *via* the gut microbiome, lowers blood pressure. Curr Hypertension Rep (2022) 24(11):509–21. doi: 10.1007/s11906-022-01216-2 PMC9568477 35838884

[25] Floch MH. The effect of probiotics on host metabolism: the microbiota and fermentation. J Clin Gastroenterol (2010) 44(1):S19–21. doi: 10.1097/MCG.0b013e3181dd4fb7 20505531

[26] Yao Y Cai X Fei W Ye Y Zhao M Zheng C. The role of short chain fatty acids in immunity, inflammation and metabolism. Crit Rev Food Sci Nutr (2020) 62:1–12. doi: 10.1080/10408398.2020.1854675 33261516

[27] Parada Venegas D de la Fuente MK Landskron G González MJ Quera R Dijkstra G. Short chain fatty acids (SCFAs)-mediated gut epithelial and immune regulation and its relevance for inflammatory bowel diseases. Front Immunol (2019) 10:277. doi: 10.3389/fimmu.2019.00277 30915065 PMC6421268

[28] Saitoh Y Suzuki H Tani K Nishikawa K Irie K Ogura Y. Tight junctions. structural insight into tight junction disassembly by clostridium perfringens enterotoxin. Science (2015) 347(6223):775–8. doi: 10.1126/science.1261833 25678664

[29] Capaldo CT Powell DN Kalman D. Layered defense: how mucus and tight junctions seal the intestinal barrier. J Mol Med (2017) 95(9):927–34. doi: 10.1007/s00109-017-1557-x PMC5548832 28707083

[30] Rodríguez-Feo JA Puerto M Fernández-Mena C Verdejo C Lara JM Díaz-Sánchez M. A new role for reticulon-4b/nogo-b in the intestinal epithelial barrier function and inflammatory bowel disease. Am J Physiol Gastrointestinal Liver Physiol (2015) 308(12):G981–93. doi: 10.1152/ajpgi.00309.2014 25907690

[31] Michielan A D'Incà R. Intestinal permeability in inflammatory bowel disease: pathogenesis, clinical evaluation, and therapy of leaky gut. Mediators Inflammat (2015) 2015:628157. doi: 10.1155/2015/628157 PMC4637104 26582965

[32] Chi JH Kim YH Sohn DH Seo GS Lee SH. Ameliorative effect of alnus japonica ethanol extract on colitis through the inhibition of inflammatory responses and attenuation of intestinal barrier disruption *in vivo* and *in vitro* . Biomed Pharmacother (2018) 108:1767–74. doi: 10.1016/j.biopha.2018.10.050 30372880

